# Machine learning‐based prediction of osteoporosis in postmenopausal women with clinical examined features: A quantitative clinical study

**DOI:** 10.1002/hsr2.1656

**Published:** 2023-10-25

**Authors:** Kainat A. Ullah, Faisal Rehman, Muhammad Anwar, Muhammad Faheem, Naveed Riaz

**Affiliations:** ^1^ Department of Computer Science and Information Technology Lahore Leads University Lahore Pakistan; ^2^ Department of Statistics and Data Science University of Mianwali Mianwali Pakistan; ^3^ Department of Information Sciences, Division of Science and Technology University of Education Lahore Pakistan; ^4^ School of Technology and Innovations University of Vaasa Vaasa Finland; ^5^ School of Electrical Engineering and Computer Science (SEECS) National University of Sciences & Technology Islamabad Pakistan

**Keywords:** classification, machine learning, osteoporosis, osteoporotic fractures, prediction

## Abstract

Osteoporosis is a skeletal disease that is commonly seen in older people but often neglected due to its silent nature. To overcome the issue of osteoporosis in men and women, we proposed an advanced prediction model with the help of machine learning techniques which can help to identify the potential occurrence of this bone disease by its advanced screening tools. To achieve more reliable and accurate results, various machine‐learning techniques were applied to the presented data sets. Moreover, we also compared the performance of our results with other existing algorithms to solely focus on the advanced features of the proposed methodology. The two data sets, the clinical tests of patients in Taiwan and medical reports of postmenopausal women in Korea through Korean Health and Nutrition Examination Surveys (2010–2011) were considered in this study. To predict bone disorders, we utilized the data about females and developed a system using artificial neural networks, support vector machines, and K‐nearest neighbor. To compare the performance of the model Area under the Receiver Operating Characteristic Curve and other evaluation metrics were compared. The achieved results from all the algorithms and compared them with Osteoporosis Self‐Assessment Tool for Asians and the results were noticeably better and more reliable than existing systems due to the involvement of ML. Using machine learning techniques to predict these types of diseases is better because physicians and patients can take early action to prevent the consequences in advance.

## 1 INTRODUCTION

In 1993, osteoporosis was discovered as a skeletal disease that is caused by low bone density,^1^, ^2^ dislocation of bone structure, and also increases the fragility and sensitivity of bones. Osteoporosis is often neglected or slightly considered around the globe but this disease has a great negative impact on the health of humans because it can cause disability and in severe cases, it can also cause mortality.^3^ Mostly in older people, this bone disease is increasing rapidly^4^ but there are no necessary preventive measures are taken which is causing higher rates of bone fractures and other vertebral diseases. The expansion rate of osteoporosis^4^ and causing fractures is increasing in more than 50‐year‐old individuals. Proper medication, precautionary measures, and maintenance of proper lifestyle can effectively reduce osteoporotic fractures,^5^, ^6^ many surveys and research have shown a ratio of 66% reduction rate in these osteoporotic patients, so timely detection of these issues can prevent other serious bone problems. It is a preventable and diagnosable disease if it is detected and diagnosed in time. According to National Osteoporotic Foundation,^7^ 50% of women and 25% of men must suffer from osteoporotic fractures their whole life. Most patients don't feel the symptoms of bone breakage until they are fractured that's why it is also called a silent disease. According to rheumatologists, millions of people are suffering from osteoporosis in America,^2^ so people should take serious consideration into their health and should get proper medication or bone tests for advanced preventive measures. The most common symptoms of osteoporosis^8^ are slight fractures issue in sensitive areas like the hip, wrists, vertebral column, and so on if a person observes a height loss of around 2 in and a curved shape of the vertebral column with noticeable weakness in the spine, these are all the warning signs for having a properly detailed checkup related to bone density and health of vertebral column. Cervical spine abnormalities can be detected easily and more accurately with the help of an accurate segmentation. In a recent research paper,^9^ a deep learning model was proposed for the segmentation of cervical vertebrae, with the ability to capture information with x‐ray images and can also eliminate noise issues for better results. Poor nutritional condition, less body weight, lack of physical activities, endocrine and cardiometabolic factors, and also age factor cause the problem of osteoporosis in females.^5^ For screening and identification of osteoporosis, many researchers have proposed several techniques for analysis of risk or causing factors as well as the methods for proper diagnosis. For instance, for postmenopausal Asian women, a method was introduced which is called the Osteoporosis Self‐Assessment Tool for Asians (OSTA) model.^10^ Other regression‐based models were also devised but machine learning techniques are gaining more popularity due to their flexible, easy‐to‐develop, and useful nature, for the detection of complex and difficult relationships between input/output data and prediction models. For classification of fractures and diagnosis of osteoporosis machine learning have shown such promising results with great accuracy. In various medical fields, machine learning approaches are being implemented for different purposes like diagnosis, detection, and risk prediction of potential fractures using different types of data sources.

Different studies and research proposed multiple methods for diagnosis, prevention, prediction, and assessment of risk factors with radiology^11^‐based methods, bone mineral density, CT scanning,^11^ and other Deep learning models^12^ as well. For prediction^13^ of bone diseases, machine learning‐based techniques hold an important position and also provide effective methods specifically focusing on the women passing from the menopausal or postmenopausal phase but these methods provide reliable results for smaller groups of people but cannot work properly with the large group of individuals. In this research, we mainly focused on aged female patients more than 50 years old and developed a prediction model^14^ with the help of machine learning algorithms to add more varsity to the scope of the subject the data of pregnant women was also considered, to show that how the weaknesses of mother's bones and other deficiencies can cause bone issues in the newborns. We used their medical, genetic, and physical characteristics as well as their laboratory test records as our three data sets from different areas for developing a well‐trained and reliable prediction model. When an automated model, based on medical and technological algorithms, is used to diagnose osteoporotic issues then the risk factors^15^ and potential problems can be reduced to half and patients having any genetic bone issue can take some precautionary measures before time.

In this paper, in the first section, we provided a thorough background of the topic with a brief introduction to the topic. We also discussed causing factors of bone diseases and their prevention methods for aged people. We mainly focused on the females having problems with their bones and bone material density because normally osteoporosis is a type of disease that show fewer symptoms until it happened. In Section 2, we proposed a method for the prediction of osteoporosis in women with supporting data sets from Taiwan, Korea, and China these data sets were the records of the patients who got their physical checkups and seem to have low bone density or weak bones. After this, we did some processing on the given data and derived some features from the medical reports of the patients, and use these characteristics to find our desired results. We compared the results of different models of machine learning on the data sets with traditional or existing models like OSTA.^7^ In Section 3, we proposed thorough and detailed models and evaluated the results, using the given features according to participants. Finally, we provided a detailed conclusion with the results of both data sets and challenges in our research following some future trends and recommendations in the field.

## 2 LITERATURE REVIEW

The risk of developing osteoporosis in people of more than 50 years is around millions of people in the United States. According to research of Yi et al.^16^ in 2002, the cost of treating osteoporotic patients was more than 14 billion US dollars. Many advanced techniques are available for early diagnosis and prevention of osteoporosis in starting phases. Different surveys and interviews are done to analyze the main causing factors which can only be done with the help of patients and their medical history. According to Kirilova et al.,^4^ the most recommended and reliable way to assess osteoporosis is the analysis of bone mineral density with dual‐energy x‐ray absorptiometry. This is a cost‐effective and moderate method but it involves an adequate amount of radiation exposure. The final report presented by Johnson et al.^10^ includes two values, one is the T‐score which means the comparison of the bone health of a person with the average young person while the Z‐value is the comparison of the bone health of an average young person with the bone condition of old people. Mostly T‐score is commonly focused on menopausal males as well as females.

In clinical settings, the most used technique is radiographic imaging which is used for many reasons like trauma assessment or pain analysis, and so on. There are multiple ways to measure bone mineral density to select postmenopausal women for research purposes. Kim et al.^17^ conducted a research to compare the advanced machine learning models with the conventional models to predict osteoporosis and the data were collected from the Korean health campaign survey Korean Health and Nutrition Examination Surveys (KNHANES) V‐1.^18^ These advanced and traditional models are evaluated and compared to analyze their best results. The ML models which are used during the study were Support Vector Machine (SVM), Random Forest (RF), Artificial Neural Network (ANN), and Logistic Regression (LR). Kim et al.^17^ presented a research and the results were in favor of SVM with Area under the Receiver Operating Characteristic Curve (AUROC) of 82.7%, an accuracy of 76.7%, a sensitivity of 77.8%, and a specificity of 76%, leaving behind the ANN, RF, LR, and traditional assessment model as well.

Nam et al.^19^ conducted a research to assess osteoporosis using Hounsfield Units of lumbar scan with data from QCT. In this study, 70 patients were going through a lumbar CT scan and 198 lumbar vertebrae were diagnosed with spine surgery. The LR method was applied to determine the T‐score using three variables (gender, age, Hounsfield units) and the T‐score of QCT also. For the prediction of osteoporotic and nonosteoporotic vertebrae, another regression model was applied using TensorFlow and Python. This multiple regression model predicted similar T‐scores. Similar T‐scores were generated with different predictive models with multiple regression algorithms. Lumbar vertebrae were classified into two groups osteoporotic and nonosteoporotic with an accuracy of 88%. In the testing of vertebrae classification by Nam et al.,^19^ the classification accuracy was 92.5%, and precision was 0.939% with the AUROC of 90%.

In 2022, a project was initialized by He et al.^20^ to gather information on radiomics from spinal or pelvic X‐rays to categorize the risk factors into “low,” “average,” and “high.” In this study, a total of 109 persons participated 38 were normal without any issues, 32 have osteopenia, and 39 have osteoporosis. The basic idea was not to replace or remove the DEXA technique but to develop a system with the qualities of proactively identifying the potential presence of osteoporosis with magnetic resonance imaging with radiomics, after selecting some common features in the participants. The AUROC results were 0.772, 0.772, and 0.778, respectively.

## 3 DATA SETS

### 3.1 Data set 1

**Figure 1:**
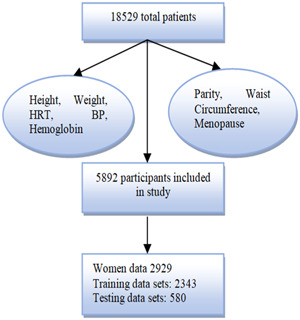
Flowchart of Data set 1 and preprocessing of patients HRT (hormone replacement therapy) BP (blood pressure).

The first data set was comprised of individuals who went through complete checkups in the medical centers in Taiwan from 2008 to 2018^7^ as shown in Figure 1. In these examinations, the medical history, family medical history, and way of living were considered they also checked the main symptoms or signs of having osteoporosis like lower back weakness, the height of patients, and their weights as well. Their test results were different but as a whole two things were required from them which were hematological and biochemical records. One important thing which is mostly considered for examining the bone's health is BMD at the lumbar spine and bilateral hip joints. Young, healthy individuals of the same gender, age, and ethnicity had their BMD findings compared to those of others in similar circumstances. The lowest T‐score was used to differentiate between osteoporotic and normal people as in the comprehensive report. The T‐score illustrates the standard deviation of healthy patients from the others.

Different features of the medical reports of patients were selected like the health of their bones, physical health, diabetes history, hypertension issues, functioning of the liver, blood flow,^16^ protein in the blood, iron concentration in bones, electrolytes, thyroid function, lipid condition, obstetrics, and most importantly gynecological records also. These medical records were thoroughly examined and analyzed to predict any bone deficiency or loss of bone density. The most common and most representative features from each domain were selected to analyze the noticeable difference between the healthy BMD group and decreased BMD group for better predictions. After the whole observation, 15 inputs were selected for females. These features included age, physical health, diabetes history, hypertension issues, functioning of the liver, parity, and protein in the blood, waist circumference, electrolytes, thyroid function,^21^ lipid condition, obstetrics, and most importantly gynecological records, menopause status, and hormone replacement therapy (HRT) records as well. Having a prior history of diagnosis and a blood pressure reading of more than 140 mm were used to define diabetes and hypertension.

### 3.2 Data set 2

**Figure 2:**
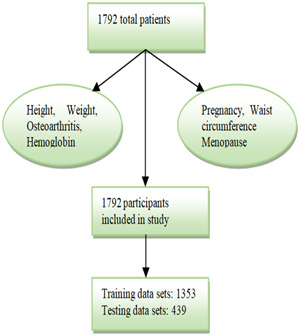
Flowchart of Data set 2 of postmenopausal women.

The second data set was KNHANES^22^ from 2010 to 2011; this data included the medical information of 1792 postmenopausal women. From the total number of participants, only 613 females were diagnosed with osteoporosis. KNHANES is a type of survey that has been conducted to analyze the health and nutritional condition and for risk assessment of the occurrence of various serious diseases. The main purpose of the study is to provide data to national organizations for analyzing health conditions and revision of policies regarding health. The data of the patients were analyzed with the R software version and preprocessing was also done to extract some common features. The whole data of KNHANES is divided into two categories like training and testing data sets (Figure 2).

### 3.3 Data set 3

From January 1, 2012, through December 31, 2021, a diagnostic investigation was conducted in a cohort of pregnant Chinese women.^23^ A total of 2560 participants who had twin or triplet pregnancies and 3159 that had missing data from any relevant characteristics were eliminated. Finally, 10802 women and their infants were taken into account in the analysis.

Before enrollment, every participant provided written informed consent, and the information gathered from them was deidentified. Early in pregnancy, participants were recruited, and they were followed up with clinical information at each visit until 1 month following delivery. The following features were observed: demographic information and previous pregnancies: age at pregnancy, ethnicity prepregnancy body mass index (BMI), parity, and uterine scarring; nutritional conditions during pregnancy anemia, deficiencies of folic acid, iron, calcium, and vitamin D complications and comorbidities such as placenta Previa, placental abruption, gestational diabetes, and gestational hyper Age at conception, prepregnancy BMI, and neonatal birth weight were among the continuous independent variables that were transformed into categorical variables to lessen the impact of extreme values.

To choose key factors, an LR analysis was first conducted to improve the computing efficiency of the ANN model. Then, using the precedent procedure, the potentially predictive factors were included in a multivariable model. Odds ratios and 95% confidence intervals (CIs) were used to determine the relationship between each component and the risk of MBD. To develop the ANN model, variables with statistical significance were reserved.

## 4 PROPOSED METHODOLOGY

For analyzing and predicting the bone disease of osteoporosis, we propose machine learning techniques like K‐nearest neighbor (KNN)^7^ and SVM^7^ and ANN^23^, ^24^ as well as various models of ANN. In this diagnostic study, five ANN models, KNN and SVM are presented with different data sets from multiple countries, were presented to provide early predictions about newborns at risk for MBD utilizing various exposure factors collected during the prenatal and/or postnatal periods. Models 1 (important prenatal and postnatal factors) and 5 (postnatal factors) performed best. The basic purpose of this study is to formulate a way to predict the existing or potential occurrence of osteoporosis but according to surveys in these data sets the expansion rate was low, 10.4% in women, and 3.8% in men, respectively.

**Figure 3:**
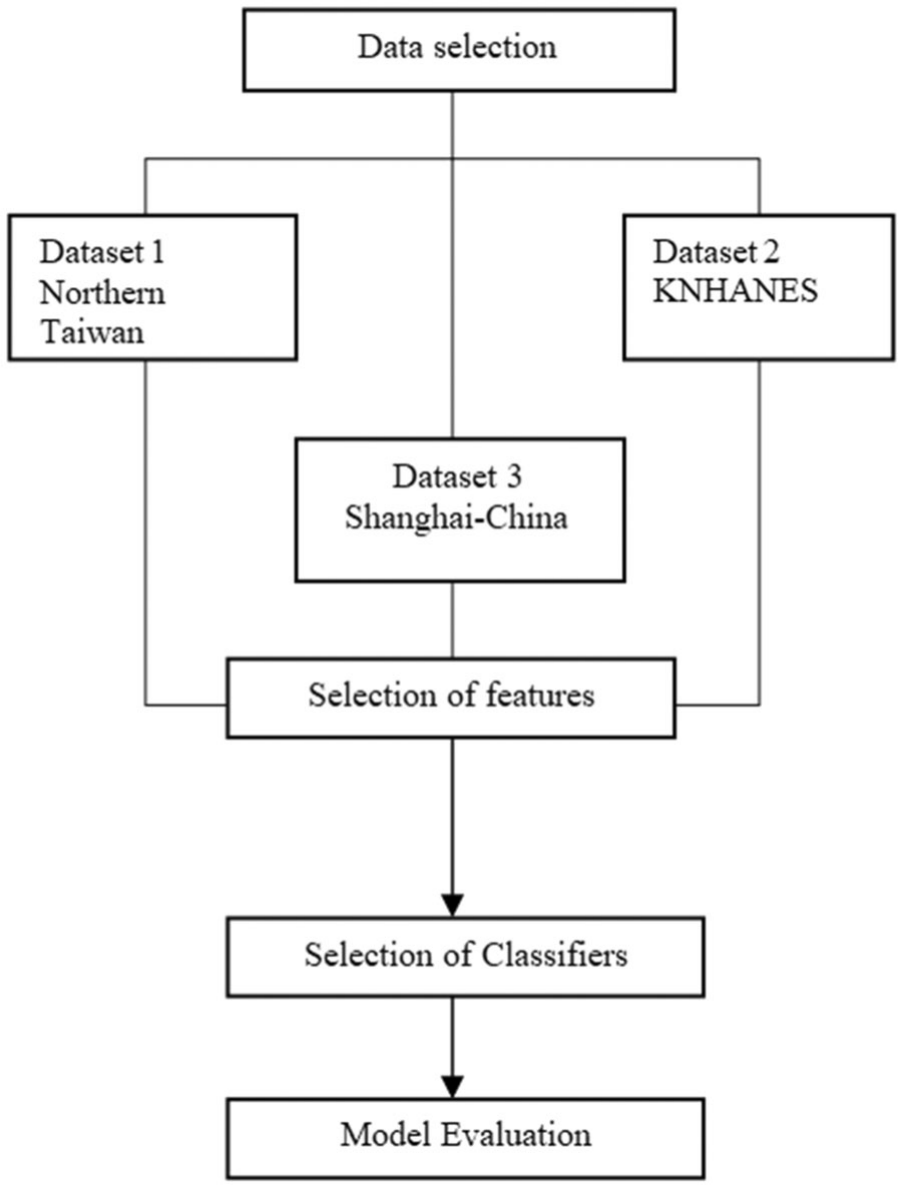
Conceptual flowchart of the proposed methodology.

This high difference in ratios can cause problems in the training of the machine learning models. The training of the model with the prediction values of BMD difference in osteopenia and osteoporotic persons can be presented by 1 and 0, respectively; they can also be used to overcome the problem of outliers. Data for patients is divided into training and testing data sets. The training data sets are rescaled for the SVM, ANN, and KNN models separately while testing sets are also rescaled with the help of the training data set index. Figure 3 represents the conceptual framework of the proposed methodology.

### 4.1 Selected classifiers

The machine learning algorithms used in the study were KNN, SVM, and ANN models developed with Tensorflow while SVM and KNN are developed with Scikit‐learn with the help of Python. Tensorflow 1.14.0 and Scikit‐learn 0.21.2 were utilized for developing the ANN model and the other models, correspondingly, in a Python 3.7 environment. The models were trained separately for best prediction, a better estimate of the expansion ratio, and a reduction of outliers. When training the data sets, we will take some trained sets for analyzing the performance of the models like SVM, ANN,^23^ and KNN in real time. On the other side, to reduce the complexity and difficulty of the models, we put a restriction on the number of iterations. For the ANN model, different parameters were tested like hyperparameters, different combinations of hidden layers, the number of nodes at each hidden layer the learning rate, and the dropout termination or rate. Different searches for all models of ML are done to analyze the best prediction model.

### 4.2 Model evaluation

For the selection of input features, the normal and the affected BDM's were compared. Some of the features hold the most importance due to their nature like HRT, Parity, and gynecological history for analyzing women's bone density while some features are not as important as others. Three machine learning models (KNN, ANN (Multiple ANN models, and SVM) and the existing OSTA model which is a self‐assessment tool for osteoporosis are compared according to their outcomes but the results showed the efficiency of the proposed model. The specificity, sensitivity, and AUROC are observed and calculated with the termination value but the prediction by ML models was much better than the OSTA model. There were also some terminations points added in the models to make the algorithms work more efficiently and these models provided 75%–92% sensitive and 61%–70% specific results for women expecting to have osteoporosis in the future.

## 5 STATISTICAL ANALYSIS

To determine the expected odds of getting osteoporosis in each model, we applied the testing data set to various models. ROC curves might be created when we tested these probabilities with the real condition set to either having or not having osteoporosis. To evaluate and contrast the effectiveness of several machine learning models, AUROC was determined. We calculated the AUROC's 95% CI and compared various AUROC values. The OSTA score was taken into account to evaluate the effectiveness of the machine learning models used in our study with that of more established models. On the testing data set, we applied the OSTA score and calculated and compared the AUROC. The weight was chosen at 0.6 to maximize sensitivity without drastically reducing specificity. At the appropriate cutoff, the sensitivity and specificity were computed.

## 6 RESULTS AND DISCUSSIONS

After performing tests and applying ML models to the data sets, we get to know that of all the study participants, 49% of women with an age ratio of more or less than 59.3 years old. The results of the Dual‐energy x‐ray showed that, from the testing data sets of 580, a 10.4% ratio of women having Osteoporosis while 46.8% are having osteopenia.

In this paper, three machine learning algorithms are proposed and applied to data sets to generate more reliable and clear results as compared to the existing models like the OSTA model. These machine learning algorithms are specifically used to perform screening on medical history of patients^10^ to predict the occurrence of osteoporosis in people more than 50 years old. There were also some terminations points added in the models to make the algorithms work more efficiently and these models provided 75%–92% sensitive and 61%–70% specific results for women expecting to have osteoporosis in the future. The ANN, SVM, and KNN models performed very accurately for the prediction of osteoporosis as compared to the existing and trusted OSTA model.

Early models for the prediction purposes of bone diseases in women used the ML technique SVM using the features of the menopausal phase, breastfeeding period, height, weight, age of the person, estrogen level, hypertension, and diabetes mellitus as input characteristics. The study^25^ demonstrated that the risk of MBD during infancy was significantly influenced by Extremely Low Birth Rate and Very Low Birth Rate.

Birth weight and gestational age were found to be the highest risk factors for MBD in preterm newborns in previous research, and it was advised that MBD screening be done on children with birthweights <1500 g. Thirty‐two infants born prematurely will partially or completely miss this crucial time of bone growth since around 80% of fetal bone mineral accretion occurs in the last 3 months of pregnancy.

Table 1 shows the selected Features of Data sets 1 and2 based on their medical health condition and their *p* value results to discriminate the osteoporotic and normal participants.

**Table 1:** Selected features of Data sets 1 and 2 based on their medical health condition and their *p* value results to discriminate the osteoporotic and normal participants.

Feature selection of the participants of Data set 1 based on their health conditions and calculation of results for normal and Osteoporotic patients				Feature selection of Data set 2 of postmenopausal women based on their medical history				
Features	Total *n* = 5982	Females *n* = 2929	*p* Value	Features	Total *n* = 1792	Training set *n* = 1343	Test set *n* = 439	*p* Value
Age (in years)	59.3 ± 7.0	59.3 ± 7.0	0.9650	Age (in years)	63 (57–70)	63 (57–70)	63 (57–70)	0.840
Diabetes history	1087 (18.0)	467 (15.6)	<0.0001	Diabetes	217 (12.1)	160 (11.8)	57 (13.0)	0.574
Hypertension	2336 (37.2)	998 (33.3)	<0.0001	Hypertension	745 (41.6)	567 (41.9)	178 (40.5)	0.655
Liver functioning	4.50 ± 0.30	13.2 ± 1.1	<0.0001	Height (cm)	154 (150–158)	153 (150–157)	155 (151–158)	0.001
Blood test	27.5 ± 18.7	24.4 ± 16.6	<0.0001	Weight (kg)	57 (52–62)	56 (52–62)	58 (53–62)	0.070
Protein In blood	55.1 ± 16.4	61.78 ± 1.57	<0.0001	Waist circumference (cm)	82 (76–88)	82 (76–88)	83 (76–88)	0.310
Electrolytes	4.50 ± 0.30	13.2 ± 1.1	<0.0002	Pregnancy	5 (3–6)	5 (3–6)	4 (3–6)	0.358
Thyroid function	27.5 ± 18.7	24.4 ± 16.6	<0.0001	Menopause duration	12.0 (5.0–21.0)	13.0 (5.0–21.0)	12.0 (5.5–21.5)	0.746
Body height	171.8 ± 8.4	165.7 ± 5.5	<0.0002	Osteoarthritis	566 (31.6)	434 (32.1)	132 (30.1)	0.467
Body weight	63.5 ± 11.7	56.9 ± 9.2	<0.0002	BMI	24.0 (22.1–26.2)	24.0 (22.1–26.2)	24.1 (22.0–26.2)	0.959
Menopause	2448 (83.6)	2448 (83.6)	<0.0001	Physical activity (*n*)	591 (33.0)	440 (32.5)	151 (34.4)	0.504
HRT	278 (9.7)	278 (9.7)	<0.0001	Past fractures	264 (14.8)	202 (14.8)	63 (14.2)	0.770
Waist circumference	83.9 ± 9.8	79.9 ± 9.5	<0.0001	Therapy of estrogen	306 (17.0)	238 (17.6)	69 (15.6)	0.364
Hemoglobin	14.1 ± 1.4	13.2 ± 1.1	<0.0002	Hyperlipidemia	379 (21.0)	273 (20.2)	104 (23.7)	0.152
Parity	2.5 ± 1.5	2.4 ± 1.4	<0.0001					

**Results**				**Results**				
Normal (*n*, %)	3058 (51.1)	1256 (42.9)						
Osteopenia (*n*, %)	1369 (46.7)	1369 (46.7)						
Osteoporosis (*n*, %)	421 (7.0)	304 (10.4)		Osteoporosis	613 (34.2)	449(33.2)	164 (37.4)	0.123

Abbreviations: BMI, body mass index; HRT, hormone replacement therapy.

The results shown by the research were 77.8% sensitivity, 76% specificity, and an AUROC of 0.767–0.811 in women.

A few years later, in 2019, another study presented a study focusing on women with the age more than 20 years in another study ANN model was specifically used and presented very reliable results. In 2020,^22^ another researcher applied several ML algorithms on 1792 data sets and provided the best performance with the ANN model. According to studies, the ratio of expansion of osteoporosis is less in women as compared to men because of the heavyweight workloads or smoking, while in women, the main reasons seen for the deficiency of bone density and osteoporosis are lack of estrogen in the postmenopausal phase and senile osteoporosis. The advantage of this study is that we mainly focused on women's osteoporosis with a large data set of 2929 and numerous advanced features, these features hold an important position for evaluating bone health in women during and after their menopausal phase.

There were some limiting factors in our paper which included some of the input features that were prone to recall bias and history taking. Second, different categories of bone density are described as normal, osteopenia, and osteoporosis through the results of dual‐energy x‐ray absorptiometry (DXA). The performance of the model could be further improved if we use T‐score as the original information. Another thing was the low prevalence rate of osteoporosis in both genders of patients 50 years old. Another thing that should be considered by the researchers is the concentration of study on different groups of people regardless of their gender and ethnicity also the validity of the ML models should be increased. From all the given models of ML, the best performance was presented by ANN (0.743) and by the models of ANN followed by SVM (0.728) and KNN (0.713) as shown in Table 2.

**Table 2:** Evaluation of several machine learning models of postmenopausal women from Taiwan and Korea with Data sets 1 and 2, respectively (Metric 1* = AUROC 95% CI, Metric 2** = sensitivity, Metric 3*** = specificity).

Evaluation of ML models with Data set 1					Evaluation of ML models with Data set 2				
Models	Metric 1* (%)	Metric 2** (%)	Metric 3*** (%)	*p* value compared with OSTA	Models	Metric 1*	Metric 2**	Metric 3***	Accuracy (95% CI)
ANN	78.2	85.5	69.7	0.0257	ANN	0.747	0.58	0.66	0.713 (0.672–0.753)
SVM	81.2	88.8	67.6	0.0142	SVM	0.0142	88.8%	67.6%	0.728 (0.674–0.758
KNN	7.68	85.5	77.5	0.0345	KNN	0.0345	85.5%	77.5%	0.743 (0.693–0.777)
OSTA	74.5	74.6	69.5						

Abbreviations: ANN, artificial neural networks; AUROC, Area under the Receiver Operating Characteristic Curve; CI, confidence interval; KNN, K‐nearest neighbor; OSTA, Osteoporosis Self‐Assessment Tool for Asians; SVM, support vector machines.

For Data set 3 from China (Table 3), there were a total of five ANN‐based models used; Model 1 incorporated important antenatal and postnatal characteristics, such as age at conception, a lack of folic acid, the addition of iron, calcium, and so on. Model 2 incorporated the mother's nutritional status parameters, such as age at conception, prepregnancy BMI, employment, parity, and so on. Model 3 took into account characteristics, gestational hypertension, use of dexamethasone, and magnesium sulfate use. Model 4 took into account every prenatal component, including the age of the pregnancy, prepregnancy BMI, occupation, parity, deficiency of folic acid, iron and calcium supplementation, gestational hypertension, dexamethasone use, and magnesium sulfate use. Neonatal birth weight, neonatal anemia, neonatal septicemia, and neonatal respiratory distress syndrome were incorporated in Model 5's postnatal factors (i.e., age at pregnancy, prepregnancy BMI, occupation, and parity).

**Table 3:** Performance evaluation of different ANN models for predictive analysis of pregnant women having the issue of BMD from Data set 3 ((Metric 1* = AUROC 95% CI, Metric 2** = sensitivity, Metric 3*** = specificity, Metric 4**** = true positive value, Metric 5***** = negative predictive value).

Evaluation of various ANN models with Data set 3						
Models	Metric 1*	Metric 2**	Metric 3***	Metric 4****	Metric 5*****	Accuracy (95%)
Model 1	0.982 (0.971–0.993)	0.952 (0.886–1.010)	0.966 (0.960–0.973)	0.260 (0.190–0.330)	0.998 (0.998–1.010)	0.97 (0.967–0.976)
Model 2	0.651 (0.572–0.724)	0.710 (0.569–0.845)	0.570 (0.553–0.587)	0.022 (0.014–0.029)	0.994 (0.991–0.998)	0.57 (0.570–0.572)
Model 3	0.818 (0.801–0.893)	0.684 (0.541–0.826)	0.890 (0.881–0.900)	0.075 (0.050–0.101)	0.996 (0.994–0.999)	0.89 (0.887–0.889)
Model 4	0.850 (0.785–0.915)	0.829 (0.714–0.944)	0.752 (0.738–0.767)	0.042 (0.029–0.055)	0.998 (0.996–1.000)	0.76 (0.754–0.756)
Model 5	0.978 (0.967–0.989)	0.951 (0.885–1.000)	0.926 (0.917–0.935)	0.142 (0.100–0.184)	0.999 (0.998–1.000)	0.93 (0.926–0.926)

Abbreviations: ANN, artificial neural networks; AUROC, Area under the Receiver Operating Characteristic Curve; CI, confidence interval.

## 7 CONCLUSION

Three machine learning^26^ models (KNN, ANN, and SVM) and the existing OSTA model which is a self‐assessment tool for osteoporosis are compared according to their outcomes but the results showed the efficiency of the proposed model. The specificity and sensitivity are observed and calculated with the termination value but the prediction by ML models was much better than the OSTA^7^ model. There were also some terminations points added in the models to make the algorithms work more efficiently and these models provided 71%–95% sensitive and 57%–92% specific results and reached AUROC of 0.767–0.978 in women. The outcome of our discussed machine learning models was almost similar with s slight differences in results but they outperform the OSTA, a traditional model for the prediction of osteoporosis. Regarding the performance of ML models, it is tough to finally draw a winner but using these models to predict the presence of osteoporosis will be a great help for the patients and physiologists. Applying the same models to the different data sets from Taiwan, Korea, and China, we got to know that ANN showed most of higher results than all other ML models.

## 8 RECOMMENDATIONS

The projected rate for patients with osteoporosis is expected to increase tremendously in the coming future. If the forecasted ratio of osteoporosis became a reality, then the next generations will face the crisis of osteoporotic fractures. But we will have new techniques and treatments to overcome this issue with the best prediction tools beforehand.

### 8.1 Early screening and diagnosis

Implement widespread screening initiatives through government health services or public health programs to find people who have poor bone density and need treatment, starting at gender‐specific ages suitable for each population and using country‐specific screening thresholds.

### 8.2 Promote creative, targeted population awareness

Spreading awareness of risk assessment initiatives with the help of accessible and targeted tools, like web‐based health screening campaigns (like the Know Your BonesTM online tool).

### 8.3 T‐notch tools and classifiers

The use of high‐class tools (such as DXA scanners), to standardize diagnostic and data‐gathering procedures is highly recommended. The data gathering system should be more appropriate and open because of the veracity and variety in data regarding age, gender, living conditions, and so on causing issues in training the data sets before actual testing. The researchers have to separately train the data of males and females due to their different selected features and living habits.

### 8.4 Precision of model

The precision of the model can only be assured if all the features regarding participants are common, so the data fields in training data sets should not be empty or missing. This thing also causes variations in the results.

According to the SCOPE study, the ratio of osteoporosis and its causing diseases will increase by 20% by 2035. As the chances of expansion of osteoporosis in the future are more than present, so researchers and field experts should tend to focus on some important aspects in the prediction of osteoporosis to get rid of it or at least control it in the earlier stages. As a whole, some things should be addressed in future research to make the prediction models work more reliably and efficiently because the accurate prediction of osteoporosis can lead to a better way of treatment beforehand.

## AUTHOR CONTRIBUTIONS

**Kainat A. Ullah**: Conceptualization; formal analysis; methodology. **Faisal Rehman**: Data curation; visualization; writing—review and editing. **Muhammad Anwar**: Project administration; resources; supervision; writing—review and editing. **Muhammad Faheem**: Funding acquisition; software; validation. **Naveed Riaz**: Formal analysis; investigation; supervision.

## CONFLICT OF INTEREST STATEMENT

The authors declare no conflict of interest.

## TRANSPARENCY STATEMENT

The lead author Muhammad Faheem affirms that this manuscript is an honest, accurate, and transparent account of the study being reported; that no important aspects of the study have been omitted; and that any discrepancies from the study as planned (and, if relevant, registered) have been explained.

## Data Availability

The data sets of KNHANES are available publically at https://knhanes.kdca.go.kr/knhanes/main.do for research purposes while the other data sets can be acquired after suitable reasons provided.
